# Transvaginal Saline Contrast Sonohystography to Investigate Postmenopausal Bleeding: A Systematic Review

**DOI:** 10.7759/cureus.10094

**Published:** 2020-08-28

**Authors:** Farshad Tahmasebi, Sarah Stewart, Anita Mitra, Mridula Morje, Ahmad Sayasneh

**Affiliations:** 1 Obstetrics and Gynaecology, Princess Royal University Hospital (PRUH), King’s College Hospital NHS Foundation Trust, London, GBR; 2 Obstetrics and Gynaecology, Institute of Reproductive and Developmental Biology, Department of Surgery & Cancer, Imperial College, London, GBR; 3 Obstetrics and Gynaecology, St Thomas’ Hospital, London, GBR; 4 Gynaecological Oncology, Guy's and St Thomas' NHS Foundation Trust, London, GBR; 5 School of Life Course Sciences, Faculty of Life Sciences and Medicine, King's College London, London, GBR

**Keywords:** saline infusion sonography, post-menopausal bleed, endometrial cancer

## Abstract

Transvaginal ultrasound (TVUS) is the initial investigation of choice for postmenopausal bleeding (PMB), followed by diagnostic hysteroscopy and endometrial sampling if abnormalities are detected. Saline contrast sonohysterography (SCSH) - injection of saline through the cervix into the uterine cavity prior to TVUS - allowed increased diagnostic accuracy in women with PMB in several small, heterogeneous studies.

The objectives of the current study were to evaluate the diagnostic accuracy of SCSH in women with PMB, comparing findings with surgical and pathological reports, highlight the necessity of SCSH in guiding clinical decision-making, and establish if there is an increase/decrease in the number of hysteroscopies performed for PMB and, hence, the adherence of clinicians to imaging referral guidelines.

The search strategy included formulating search terms identifying all synonyms of SCSH and postmenopause. The databases searched were MEDLINE, Embase, and the Cochrane Library.

Only studies comparing SCSH to an alternative method were selected. The studies were screened and data analysis performed using content analysis. Data reduction was performed through systematic coding and the generation of themes

We identified 18 studies, comprising 974 women, using SCSH to evaluate the endometrial cavity in women with PMB; most support SCSH improving diagnostic accuracy through delineating intracavitary structures.

In effect, SCSH could be a first-line investigative modality to assess the uterine cavity once a larger, well-designed study has been conducted to clarify its specificity, sensitivity, and positive predictive value (PPV). Owing to its relatively non-invasive nature and potentially high diagnostic accuracy, SCSH could allow for more accurate decisions regarding the need for further investigation and subsequent management.

Tweetable abstract

"Saline contrast sonohysterography improves the diagnostic accuracy of the endometrium in postmenopausal bleeding."

## Introduction and background

Transvaginal ultrasonography (TVUS) significantly improved the accurate diagnosis of intrauterine abnormalities. In women with postmenopausal bleeding (PMB), a measurement of endometrial thickness (ET) reliably distinguishes a women’s risk of endometrial cancer. An ET of ≤4 mm decreases the likelihood of endometrial cancer by 10-fold in users and non-users of hormone replacement therapy [1-2]. In high‐risk women (ET > 5 mm), the evaluation of endometrial morphology and vascularisation using gray‐scale and Doppler ultrasound imaging refines endometrial cancer risk [3-4]. Most studies reporting on ultrasonography of the uterine cavity are small, with sometimes conflicting results regarding sonohysterography, potentially due to heterogeneity in study design and population. Large multicentre studies are necessary to clarify the role of sonohysterography in the assessment of endometrial morphology and vascularisation to differentiate endometrial and intracavitary pathologies.

Abnormal uterine bleeding (AUB), defined as either metrorrhagia, vaginal bleeding separated from expected menses or menorrhagia, subjective complaints of either increased duration and/or volume [5]. In peri-menopausal women, variations in bleeding may be secondary to physiological hormonal changes or neoplastic changes, either benign or malignant.

Postmenopausal bleeding (PMB), or bleeding occurring >12 months after menopause, occurs immediately after menopause in approximately 10% of cases [6], signaling endometrial carcinoma in around 10% of cases [7-8], or benign conditions, such as endometrial polyps, in 20%-40% [9]. Endometrial carcinoma is the most common gynecological malignancy, and 95% of women present with PMB [10-11]. Endometrial cancer often presents at an early stage, allowing curative surgical intervention; therefore, early, accurate diagnosis is paramount.

Historically, investigation of PMB was through dilatation and curettage (D&C) [12]. Ultrasonography reduced investigatory invasiveness. Endometrial biopsy and hysteroscopy have almost completely replaced D&C. Outpatient endometrial biopsy reduces costs in diagnostic work-up, without affecting life expectancy [13]. Despite numerous studies investigating PMB, a consensus regarding the most accurate and efficient diagnostic pathway remains elusive [2,14-15].

TVUS may visualize the endometrium in postmenopausal women poorly, and distortion of the cavity by pathological abnormalities can make assessment difficult. Such limitations have led to a growing interest in saline contrast sonohysterography (SCSH).

SCSH involves the instillation of sterile saline, a negative contrast agent, into the uterus through a hysterosalpingography catheter prior to TVUS. Uterine cavity distension is ‘optimal’ if fluid clearly distends the cavity, ‘suboptimal’ if the cavity is minimally distended, and ‘failed’ if no fluid is observed within the cavity.

Compared to TVUS, SCSH has been reported in premenopausal women to allow easier differentiation of polyps, submucous fibroids, and endometrial lesions that emerge clearly in anechoic saline [16-17]. Fortunately, it requires widely available, inexpensive equipment, and has good reported diagnostic performance in the bleeding uterus [18-19].

## Review

Materials and methods

The electronic bibliographic databases, MEDLINE, Embase, and the Cochrane Library, were searched (Cochrane Database of Systematic Reviews, Cochrane Central Register of Controlled Trials (CENTRAL), Cochrane Methodology Register) on the accuracy of SCSH and other diagnostic tools in women with PMB using Medical Subject Headings (MeSH) and text words for SCSH (SCSH; Saline Infusion Sonography, Saline Infusion*; Saline Infusion Hysterosonography, Saline Contrast Sonohysterography)’, and ‘post-menopause’ (post-menopause*, menopause, post-menopause). We used the PICO model for the search strategy (Patient: post-menopausal, Intervention: saline infusion sonography, saline contrast sonohysterography, Comparison: other diagnostic modalities (outpatient/inpatient hysteroscopy or endometrial biopsy), Outcome: accuracy of saline contrast sonohysterography). The search strategy was adapted for use with other bibliographic databases in combination with database-specific filters for controlled trials, where available. Studies published from January 1980 to 2017 were considered.

Only texts in English were included. The reference lists of all known primary and review articles were examined for relevant citations not captured by electronic searches. Studies directly comparing the diagnostic accuracy of SCSH and other diagnostic modalities in women with PMB were included. Studies reporting a single technique were excluded.

Exclusion criteria included hormone therapy within the last six months, previous abnormal endometrial biopsy, cervical pathology on speculum examination, abnormal cervical smear, and history or evidence suggestive of active pelvic infection.

Content analysis was chosen as the data analysis method because of its strengths in methodically cataloging and summarizing substantial amounts of data and text. The overall approach to this method of analysis was the interpretation of patterns occurring within texts, particularly with the context of the sample data. Thus, the research coded the data in three phases: (1) absorption in the text and data, (2) reduction of the data through systematic coding and generation of themes, and (3) interpretation of findings. Using a deductive approach, data analysis was initiated with a preconceived coding template, organized according to an existing structure. Such a structure was premised on the purpose of this systematic review, to evaluate the diagnostic accuracy of SCSH in the evaluation of intracavitary abnormalities in postmenopausal women with AUB. Thus, the results of the content analysis were qualitative, summarizing key findings both by patient population and by recommendation of SCSH as a diagnostic tool.

**Figure 1:**
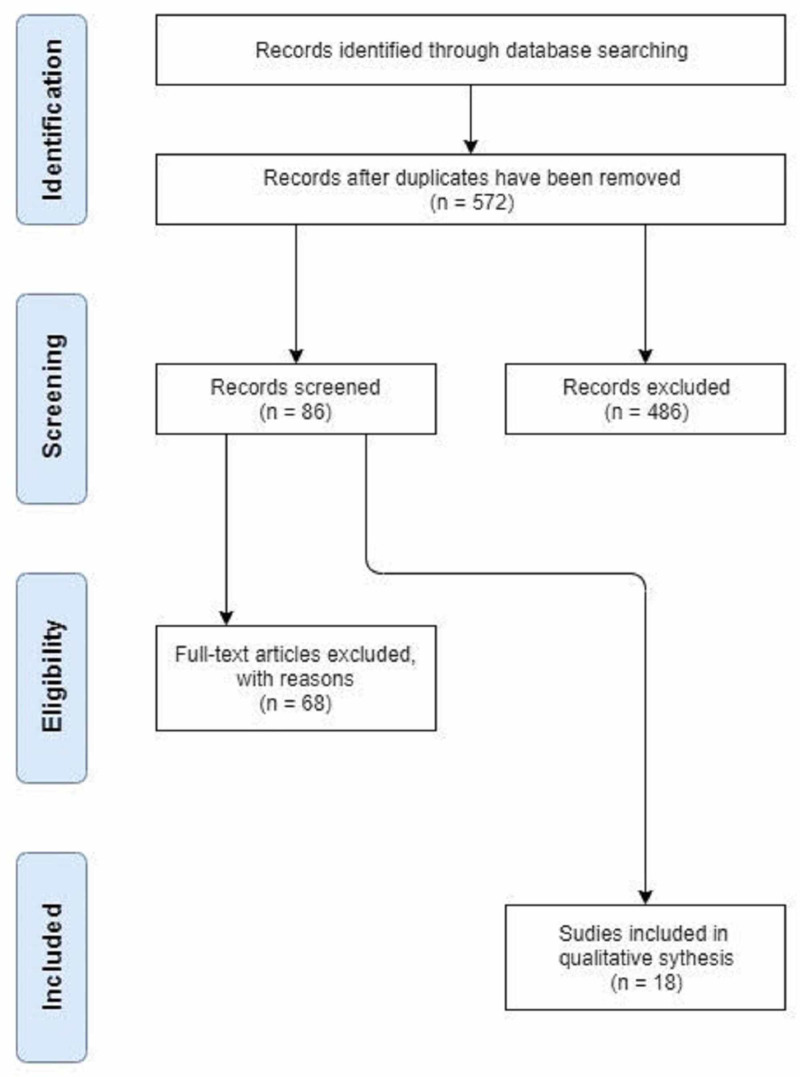
Identification and method

Figure 1 shows the process of identifying studies and the methods used.

Results

We included 18 studies comprising 974 women in this systematic review. SCSH was considered a valuable diagnostic tool for PMB [20-22] and advocated as an initial test rather than hysteroscopy to triage patients with PMB, SCSH could be the sole initial test, with endometrial biopsy used only for patients with a symmetrically thickened endometrium [23]. However, one study argued that the feasibility of using SCSH as compared with diagnostic hysteroscopy is low in postmenopausal women [24].

Others promoted SCSH in combination with TVUS as an initial diagnostic step for women with PMB [25-26] showing SCSH improves the diagnostic accuracy of TVUS through the better visualization of small intracavitary tumors; however, their populations were mostly perimenopausal women [27-28].

Miller et al. [29] and Mihm et al. [30] claimed that SCSH is valuable for patient selection and procedure planning for hysteroscopy, but did not define their population by menopausal status, therefore, these studies may not advocate SCSH in a specifically postmenopausal group. Takac et al. argued that SCSH is not a superior diagnostic tool to TVUS, a conclusion which contradicts other studies [31].

Overall, the consensus is that SCSH is a useful initial diagnostic tool to investigate PMB, but a consensus is lacking regarding its sensitivity, specificity, and positive predictive value (PPV) for detecting uterine pathology.

Postmenopausal Population

Of the 18 studies analyzed, eight focused primarily on postmenopausal women.

Evaluation 1: SCHS as equal or superior to hysteroscopy, TVUS, and D&C: Epstein et al. evaluated 105 women with PMB and ET >5 mm, comparing hysteroscopy, TVUS, and SCSH [20]. The study suggested that SCSH could replace hysteroscopy for diagnosing focal intrauterine lesions in women with PMB and SCSH was superior to TVUS at distinguishing between benign and malignant endometria. They found a 96% correlation between SCSH and hysteroscopy at identifying focal lesions, with 80% sensitivity for polyps. A conventional ultrasound had 49% sensitivity, with a false-positive rate of 19%. When discriminating between benign and malignant lesions, hysteroscopy (84%) was more successful than SCSH (66%) and conventional ultrasound (40%). Neither hysteroscopy nor SCSH could dependably differentiate between benign and malignant focal lesions, with SCSH failing in almost 20% of cases; two-thirds of this failure was attributed to cervical stenosis and another one-quarter to problems with backflow or distension.

Goldstein et al. concurred SCSH is as accurate a diagnostic tool as hysteroscopy and frequently more effective than TVUS [5]. This article was a summation of a panel discussion, not a quantitative study. Despite this limitation, the panel was monolithic in its assessment of SCSH as the appropriate tool to diagnose a focal abnormality, highlighting its safety, ease of the procedure, and equal detection rates for focal abnormalities with hysteroscopy. They noted that SCSH could be used to distinguish the nature of the focal abnormality and hysteroscopy would be used to remove it. They advocated SCSH for triaging patients, distinguishing diffuse and focal endometrial thickening, and concurring that SCSH was more sensitive than TVUS or endometrial biopsy (EMB) at detecting focal abnormalities in women with PMB.

Karsidag et al. argued that SCSH is a superior diagnostic tool to blind D&C and TVUS, with equal accuracy to hysteroscopy in diagnosing focal intrauterine lesions in 36 women with recurrent PMB following normal D&C [22]. Results showed blind D&C had 47% sensitivity and 68% specificity for diagnosing focal intracavitary abnormalities, with 57% PPV and 59% negative predictive value (NPV) while missing 65% of polyps and submucous fibroids. TVUS, had a sensitivity of 63%, a specificity of 78%, a PPV of 89%, and an NPV of 41%. TVUS failed to identify 10 patients with polyps and misdiagnosed two normal patients with polyps. SCSH had a sensitivity of 93%, a specificity of 56%, a PPV of 86%, an NPV of 71%, and a diagnostic accuracy of 83%. When comparing SCSH with hysteroscopy, sensitivity was 88%, specificity was 75% while PPV was 97% and NPV was 43%. This concluded that SCSH and hysteroscopy are equally valuable diagnostic tools, but SCSH cannot reliably discriminate between benign and malignant focal lesions. Conversely, SCSH can distinguish between the endometrium and myometrium, making it more appropriate for classifying the degree of fibroid extension.

Takac et al. were outliers arguing that SCSH is not superior to TVUS [31]. Fifty-three postmenopausal women with endometrial cancer were examined preoperatively with TVUS and SCSH to assess myometrial invasion. No statistically significant difference in the accuracy of SCSH (85%) as compared to TVUS (81%) was seen when correlated with definitive histopathologic findings. Regarding endometrioid adenocarcinomas, TVUS correctly predicted myometrial invasion in 85% as compared to SCSH 80%. SCSH had better specificity (72%) than TVUS (76%) and better sensitivity (96%) than TVUS (86%). Both modalities demonstrated 83.3% accuracy when predicting the depth of myometrial invasion in 15 cases of G1 tumors.

All four studies agreed that SCSH is a useful diagnostic tool that is easier, more cost-efficient, less invasive, and as effective as hysteroscopy. Three of four found SCSH superior to TVUS, D&C, and EMB, advocating it as an initial triage for patients with PMB.

Evaluation 2: SCSH in conjunction with TVUS: Two studies recommended SCSH in conjunction with TVUS; one assessed the combination as comparable to hysteroscopy, the other judged SCSH with TVUS as superior to EMB.

Inal et al. tested the diagnostic efficacy of TVUS and SCSH in the evaluation of uterine cavities in 60 tamoxifen-treated, asymptomatic, postmenopausal breast cancer patients [8]. TVUS and SCSH failed in four (6.7%), detected a normal endometrium in 38 (63.3%) (hysteroscopy: 66.7%), and polypoid structures in 18 (30%) (hysteroscopy: 33.3%). When compared to hysteroscopy, TVUS with SCSH had 90% sensitivity, 100% specificity, 100% PPV, and 95% NPV.

Dubinsky et al. compared TVUS and SCSH with EMB, in 81 postmenopausal women with ET > 5 mm [7]. TVUS and SCSH confirmed 45 lesions: 23 pedunculated endometrial masses (16 endometrial polyps, 4 fibroids, and 3 carcinomas) and 22 inhomogeneous sessile lesions. Of these 45 cases, 41 had false-negative aspiration biopsies. Researchers noted TVUS and SCSH failed in only eight of 89 cases, primarily due to cervical stenosis. Researchers, therefore, advocated TVUS and SCSH as an initial diagnostic tool for postmenopausal women. However, women with a positive diagnosis subsequently underwent hysteroscopy or hysterectomy, emphasizing the role and limitations of SCHS.

Evaluation 3: SCHS with endometrial biopsy: O’Connell et al. compared the combined diagnostic reliability of SCHS and EMB with fractional curettage and hysteroscopy for the initial evaluation of women with PMB [5]. The combination of SCSH and EMB positively correlated with the surgical findings in > 95% (sensitivity 94%, specificity 96%, CI: 91% - 99%). Furthermore, no patients with endometrial hyperplasia or cancer were misdiagnosed. SCSH alone had a 92% positive correlation, however, sensitivity was conspicuously hindered by the omission of EMB. Therefore, the combination was recommended for initial evaluation, noting SCSH could be the sole initial triage, with EMB used for patients with symmetrically thickened endometrium.

Outlier: outcome-based study: Meng et al. looked at the clinical outcomes of 119 women with PMB who underwent SCSH [32]. Findings indicated a statistically significant connection between a positive SCSH and more aggressive treatment; a negative SCSH resulted in more conservative treatment in 95%. Of the 80 women with a positive SCSH, abnormalities included polyps (42), submucosal fibroids (6), endometrial thickening (8), >2 of the above (7), or other; debris, adenomyosis, or indeterminate findings (17).

Perimenopausal Population

This systematic review focuses on postmenopausal women, however, one study of 159 perimenopausal patients with AUB was included [33]. It concurs SCSH is reliable at investigating AUB and differentiating the need for medical therapy from surgery. SCSH was highly sensitive (98.9%) and specific (76.4%) at distinguishing between women with intrauterine lesions from those with normal or atrophic endometrium. SCSH was accurate in the diagnosis of submucosal fibroid (sensitivity 87.8%, specificity 95%) and polyps (sensitivity 89.6%, specificity 90.7%). Although SCSH recognized only 40% of endometrial cancers, all these patients had abnormalities during SCHS, indicating a zero false‐negative rate.

Combination of Pre-, Peri-, and Postmenopausal Populations

Four studies with mixed patient populations were included. While these studies evaluations are valuable, ascertaining the applicability of their findings exclusively to postmenopausal women is difficult.

Evaluation 1: SCHS as an accurate diagnostic tool (with caveats): Two studies argued that SCSH was a more effective diagnostic tool than TVUS. Yildizhan et al. compared the diagnostic efficacy of TVUS and SCSH [6]. They showed that SCSH is more effective than TVUS. TVUS cannot distinguish between polyps, submucosal fibroids, and hyperplasia. They also failed to identify the exact location of growths and polyps. However, the authors were able to comment on the distinction between pre- and postmenopausal patients, arguing that while the feasibility of using SCSH in premenopausal women is comparable with diagnostic hysteroscopy, it is low in postmenopausal women. The authors recommend EMB as the primary method for diagnosing endometrial hyperplasia and carcinoma in postmenopausal women.

Dubinsky et al. included 88 pre-, peri-, and postmenopausal women [34]. The authors concluded SCSH was an appropriate, effective diagnostic tool but not as valuable or suitable for detecting carcinoma. The PPV for carcinoma was 16%, suggesting that women with multifocal or sessile lesions should undergo guided biopsy, and benign-appearing polyps should be removed to control bleeding and eliminate the risk of carcinoma in situ. However, using SCSH, women with a benign endometrial disease could seek medical therapy, avoiding invasive diagnostic procedures. The inclusive patient population makes it unclear if results can be connected to women’s menopausal status.

Evaluation 2: SCHS in combination with TVUS: Schwarzler et al. argued that SCSH improves the diagnostic accuracy of TVUS through the better visualization of small intracavitary tumors [27]. Studying 100 patients, researchers found that the sensitivity and NPV of TVUS (67% and 71%, respectively) improved considerably (87% and 86%, respectively) with SCSH. Moreover, the number of false-negative findings for this type of pathology reduced significantly. The addition of SCSH to TVUS did not significantly enhance the detection of malignant and premalignant changes in the endometrium. Moreover, a sizeable proportion of the study population were premenopausal women.

Pasrija et al. studied 58 women, finding that SCSH enhanced the diagnostic accuracy of TVUS [28]. Results indicated that TVUS alone had 84.8% sensitivity, 79% specificity, 82.4% PPV, and 82% NPV. The addition of SCSH increased sensitivity to 94.1%, specificity to 88.5%, PPV to 91.4%, and NPV to 92%. When compared to hysteroscopy or hysterectomy, SCSH findings missed one endometrial polyp and one endocervical polyp, as well as diagnosed a false-positive growth. Importantly, 89.6% of women were perimenopausal.

Population Unknown

This is the final category of two studies that did not define their population’s menopausal status.

Evaluation 1: SCHS should be used in certain cases: Miller et al. argued SCSH should be the diagnostic tool when TVUS shows normal ET or if endometrial biopsy findings are benign [29]. Ultimately, SCSH is a sensitive (95%) method for detecting focal intracavitary abnormalities and is valuable for patient selection and procedure planning for hysteroscopy.

Evaluation 2: SCSH with EMB:* *Mihm et al. studied 113 women, finding the combination of EMB and SCSH had 97% sensitivity and 70.2% specificity for the detection of abnormal pathologic features, with 82.1% PPV and 94.3% NPV as compared to hysteroscopy/curettage or hysterectomy [30]. Researchers argued that the combination of EMB and SCSH allows patients to circumvent more aggressive procedures, offering a more conservative treatment option. Authors acknowledged not stratifying by menopausal status, could alter treatment decisions.

Discussion

Main Findings

PMB is estimated to account for 5%-10% of gynecologic visits, which is an increasing trend due to the increased prevalence of HRT. Thirty percent (30%) of PMB is secondary to exogenous estrogen, another 30% due to atrophic endometritis or vaginitis, while 40% is due to endometrial hyperplasia (10%), endometrial polyps (10%), submucous fibromyomas (10%), and uterine malignancies (10 %). Most cancers causing PMB are endometrial in origin, accounting for 5%-8% of all PMB cases [35].

SCSH has been proposed as a means for the nonsurgical identification of intracavity abnormalities [25]. Wolman et al. reported a prospective double-blind study of 47 postmenopausal patients evaluated first by SCSH and then hysteroscopy [36]. SCSH proved accurate in the diagnosis of intraluminal masses with 86% sensitivity and 87% specificity.

The evaluation of PMB is evolving as newer imaging techniques like SCSH gain acceptance. Many algorithms for the evaluation of PMB have been introduced, most suggest starting with TVUS to assess ET, followed by SCSH to define abnormalities prior to surgical intervention or biopsy.

Numerous studies have shown ET ≤5 mm on TVUS has a high NPV for endometrial carcinoma, virtually excluding significant pathology. However, TVUS does not adequately define focal lesions.

SCSH is consistently reported as significantly better or equal to TVUS in defining intrauterine abnormalities. With several small studies reporting the diagnostic accuracy of SCSH in an exclusively postmenopausal population, ee sought to systematically analyze this data.

Strengths and limitations

In this review of 18 studies, including 974 women evaluating the use of SCSH to investigate postmenopausal bleeding, the sensitivity in individual studies ranged from 67.7%-97%, specificity from 46%-100%, with PPV ranging from 16-100 [2-4].

It was also observed that in a sub-analysis of one study, including 159 peri-menopausal women with AUB, SCSH was highly sensitive (98.9%) and specific (76.4%) in the distinction between women with intrauterine lesions and those with normal or atrophic endometrium (98.9% and 76.4%, respectively). SCSH was also accurate in the diagnosis of submucosal fibroid (sensitivity 87.8%, specificity 95%) and polyps (sensitivity 89.6%, specificity 90.7%) [37]. The review showed SCSH was in near-perfect agreement (96%) with hysteroscopy in the diagnosis of focally growing lesions. Similarly, Epstein et al. (2001), in diagnosing endometrial polyps, both SCHS and hysteroscopy had a sensitivity of approximately 80% while conventional ultrasound was 49% with a false-positive rate of 19% [15]. However, some authors found hysteroscopy (84%) was more successful than SCHS (44%) and conventional ultrasound (60%) in discriminating between benign and malignant lesions. Results were split between specificity (76% with TVUS and 72% with SCHS) and sensitivity (86% with TVUS and 96% with SCHS) [2,6].

Likewise, findings were similar in predicting the depth of myometrial invasion in 15 cases (83.3%) of G1 tumors by both SCHS and TVUS. Takac et al. [31] and Goldstein et al. [20] similarly concurred that SCHS is an equally effective diagnosis tool as hysteroscopy and frequently a more effective diagnostic tool than TVUS. Table 1 summarizes all the evidence found during our research while Table 2 lists all the studies included in the research.

**Table 1:** Summary of evidence PPV: positive predictive value

Paper	Final No. of patients	Sensitivity	Specificity	PPV
Transvaginal hysterosonography: comparison with biopsy in the evaluation of postmenopausal bleeding	148	Unknown	Unknown	Unknown
Saline contrast sonohysterography as the first-line investigation for women with uterine bleeding	Unknown	76.525	95.325	Unknown
An evaluation of sonohysterography and diagnostic hysteroscopy for the assessment of intrauterine pathology	98	87	91	92
Triage of abnormal postmenopausal bleeding: A comparison of endometrial biopsy and transvaginal sonohysterography versus fractional curettage with hysteroscopy	100	94	96	96
Saline infusion sonography (SCSH) or office hysteroscopy: Which one is the best? A prospective randomized study	53	Unknown	Unknown	Unknown
Prediction of benign and malignant endometrial disease: hysterosonographic pathologic correlation	Unknown	89	46	16
Transvaginal sonography, saline contrast sonohysterography and hysteroscopy for the investigation of women with postmenopausal bleeding and endometrium > 5 mm	105	67.7	Unknown	71.3
Evaluation of the woman with postmenopausal bleeding	N/A	N/A	N/A	N/A
The accuracy of endometrial biopsy and saline sonohysterography in the determination of the cause of abnormal uterine bleeding	Unknown	97	70.2	82.1
Saline contrast hysterosonography in abnormal uterine bleeding: a systematic review and meta-analysis	2278	95	88	LR + = 8.23
Prospective study of saline infusion sonohysterography in the evaluation of perimenopausal and postmenopausal women with abnormal uterine bleeding	Unknown	94.1	88.5	91.4
Diagnostic accuracy of sonohysterography in the evaluation of uterine cavities in tamoxifen-administered asymptomatic postmenopausal breast cancer patients with endometrial thickness ≥5 mm	60	90	100	100
The short-term clinical outcomes after saline infusion sonohysterography in women with postmenopausal bleeding	119	Unknown	Unknown	Unknown
Transvaginal ultrasonography with and without saline infusion in the assessment of myometrial invasion of endometrial cancer	53	72	96	95
Transvaginal ultrasonography and saline infusion sonohysterography for the detection of intrauterine lesions in pre- and post-menopausal women with abnormal uterine bleeding	104	91.45	95.9	93.45
Ultrasound and sonohysterography in the evaluation of abnormal vaginal bleeding	N/A	N/A	N/A	N/A
Transvaginal sonography, sonohysterography, and hysteroscopy for investigation of focal intrauterine lesions in women with recurrent postmenopausal bleeding after dilatation & curettage	36	93	56	86
A prospective comparison of transvaginal ultrasound, saline infusion sonohysterography, and diagnostic hysteroscopy in the evaluation of endometrial pathology	98	91.8	60	LR + = 2.29

**Table 2:** Summary of included studies

Paper	Year	Authors		Final No. of patients		Pre-meno No. of patients		Post-meno No. of patients	
Transvaginal hysterosonography: comparison with biopsy in the evaluation of postmenopausal bleeding	1995		Dubinsky et al. [25]		148		0		148
Saline contrast sonohysterography as the first-line investigation for women with uterine bleeding	1997		Bernard et al. [33]		Unknown		109		53
An evaluation of sonohysterography and diagnostic hysteroscopy for the assessment of intrauterine pathology	1998		Schwarzler et al. [27]		98		70		28
Triage of abnormal postmenopausal bleeding: A comparison of endometrial biopsy and transvaginal sonohysterography versus fractional curettage with hysteroscopy	1998		O'Connell et al. [23]		100		0		100
Saline infusion sonography (SCSH) or office hysteroscopy: which one is the best? A prospective randomized study	1998		Timmermans et al. [9]		53		0		53
Prediction of benign and malignant endometrial disease: hysterosonographic pathologic correlation	1995		Dubinsky et al. [34]				88		
Transvaginal sonography, saline contrast sonohysterography, and hysteroscopy for the investigation of women with postmenopausal bleeding and endometrium > 5 mm	2001		Epstein et al. [20]		105		0		105
Evaluation of the woman with postmenopausal bleeding	2001		Goldstein et al. [21]						
The accuracy of endometrial biopsy and saline sonohysterography in the determination of the cause of abnormal uterine bleeding	2002		Mihm et al. [30]				113		
Saline contrast hysterosonography in abnormal uterine bleeding: A systematic review and meta-analysis	2003		de Kroon et al. [38]		2278		2278		
Prospective study of saline infusion sonohysterography in the evaluation of perimenopausal and postmenopausal women with abnormal uterine bleeding	2004		Pasrija et al. [28]				52		6
Diagnostic accuracy of sonohysterography in the evaluation of uterine cavities in tamoxifen administered asymptomatic postmenopausal breast cancer patients with endometrial thickness ≥ 5 mm	2006		Inal et al. [26]		60		0		60
The short-term clinical outcomes after saline infusion sonohysterography in women with postmenopausal bleeding	2006		Meng et al. [32]		119		0		119
Transvaginal ultrasonography with and without saline infusion in assessment of myometrial invasion of endometrial cancer	2007		Takac et al. [31]		53		0		53
Transvaginal ultrasonography and saline infusion sonohysterography for the detection of intrauterine lesions in pre- and post-menopausal women with abnormal uterine bleeding	2008		Yildizhan et al. [24]		104		79		25
Ultrasound and sonohysterography in the evaluation of abnormal vaginal bleeding	2008		Miller et al. [29]						
Transvaginal sonography, sonohysterography, and hysteroscopy for investigation of focal intrauterine lesions in women with recurrent postmenopausal bleeding after dilatation & curettage	2010		Karsidag et al. [22]		36		0		36
A prospective comparison of transvaginal ultrasound, saline infusion sonohysterography and diagnostic hysteroscopy in the evaluation of endometrial pathology	2010		Grimbizis et al. [39]		98		77		21

## Conclusions

In conclusion, we suggest that SCSH could be used as the first-line investigative modality to evaluate the uterine cavity in PMB, owing to its relatively non-invasive nature and potentially high diagnostic accuracy to allow for more accurate decisions about the need for further investigation and subsequent preoperative planning. In cases where an endometrial lesion is detected, SCSH can act as a multipurpose investigation to both diagnose and triage the patient, assisting in decision-making regarding the need for further invasive surgical management. Hence, the role of SCHS should be to triage patients for more or less invasive therapy where an endometrial lesion is detected. It also assists in choosing the best conservative surgical treatment for the patient. In view of the heterogeneity of results obtained from the 18 studies included in this systematic review, we propose that a larger, well-designed study to evaluate the specificity, sensitivity, PPV, and NPV is required prior to the potential integration of SCSH into new guidelines for the investigation of PMB.
